# TUFT1 promotes metastasis and chemoresistance in triple negative breast cancer through the TUFT1/Rab5/Rac1 pathway

**DOI:** 10.1186/s12935-019-0961-4

**Published:** 2019-09-23

**Authors:** Weiguang Liu, Jianjun Han, Sufang Shi, Yuna Dai, Jianchao He

**Affiliations:** Department of Breast Surgery, Affiliated Hospital of Hebei University of Engineering, Handan, 056000 Hebei China

**Keywords:** Triple negative breast cancer, TUFT1, Rab5, Metastasis, Chemoresistance, Prognosis

## Abstract

**Background:**

Triple negative breast cancer (TNBC) is a breast cancer (BC) subtype that is characterized by its strong invasion and a high risk of metastasis. However, the specific mechanisms underlying these phenotypes are unclear. TUFT1 plays an important role in BC and impacts the proliferation and survival of BC cells. Recent studies have shown that TUFT1 mediates intracellular lysosome localization and vesicle transport by regulating Rab GTPase, but the relevance of this activity in TNBC is unknown. Therefore, our aim was to systematically study the role of TUFT1 in the metastasis and chemoresistance of TNBC.

**Methods:**

We measured TUFT1, Rab5-GTP, and Rac1-GTP expression levels in samples of human TNBC by immunohistochemistry (IHC) and conducted univariate and multivariate analyses. shRNA-mediated knockdown and overexpression, combined with transwell assays, co-immunoprecipitation, a nude mouse xenograft tumor model, and GTP activity assays were used for further mechanistic studies.

**Results:**

TUFT1 expression was positively correlated with Rab5-GTP and Rac1-GTP in the TNBC samples, and co-expression of TUFT1 and Rab5-GTP predicted poor prognosis in TNBC patients who were treated with chemotherapy. Mechanism studies showed that TUFT1 could activate Rab5 by binding to p85α, leading to activation of Rac1 through recruitment of Tiam1, and concurrent down-regulation of the NF-κB pathway and proapoptotic factors, ultimately promoting metastasis and chemoresistance in TNBC cells.

**Conclusions:**

Our findings suggest that the TUFT1/Rab5/Rac1 pathway may be a potential target for the effective treatment of TNBC.

## Background

Triple negative breast cancer (TNBC), a breast cancer (BC) subtype that lacks estrogen and progesterone receptor expression and has no epidermal growth factor receptor 2 amplification, accounts for about 20% of all breast cancers [1]. TNBC usually presents with less favorable clinical features than other BC subtypes, and TNBC tumors proliferate faster, relapse earlier, metastasize more frequently, and are usually associated with poorer prognosis [2]. However, the mechanism underlying the frequent metastasis of TNBC is unclear. Currently, there are no effective targeted drugs for TNBC. Therefore, cytotoxic chemotherapy is the main treatment modality [3]. In-depth studies of the mechanism underlying the metastasis of TNBC may reveal its targets and provide theoretical support for the exploration of new therapeutic drugs.

Tuftelin (TUFT1) is an acidic, hydrophilic, glycosylated, and phosphorylated protein, and analyses have shown that TUFT1 is well conserved, with high homology across species. TUFT1 plays an important role in enamel mineralization and is involved in the interaction between the mesenchymal ectoderm and autosomal enamel dysplasia during tooth development [4]. Zhou et al. [5] demonstrated that TUFT1 protein is highly expressed in pancreatic cancer and that its expression correlates with both disease stage and local lymph node metastasis. They proposed that TUFT1 may affect HIF1 by influencing the expression of components of the Snail signaling pathway, which regulates epithelial mesenchymal transition. Recent studies have shown that TUFT1 promotes the proliferation, metastasis, and epithelial-mesenchymal transformation of hepatocellular carcinoma cells through the Ca^2+^/PI3 K/AKT pathway. In addition, hypoxia increased TUFT1 expression by down-regulating the expression of miR-671-5p [6]. We previously showed that inhibition of TUFT1 expression in BC cells inhibits their proliferation, affects the cell cycle, and induces apoptosis. In addition, we showed that inhibiting TUFT1 expression altered the gene expression of Rab5, Rac1, p65, and caspase 3 [7, 8].

Rab5, a member of the Rab family of GTPases, is responsible for regulating the early stage of vesicle transport. Once activated, Rab5 recruits a number of interacting proteins, including Rac1 and Tiam1, which play an important role in vesicle transport, cytoskeletal remodeling, and tumor metastasis [9, 10]. In a recent study, Kawasak et al. [11] found that TUFT1 activated the mTORC1 signaling pathway by regulating Rab GTPase and that the interaction between TUFT1 and RabGAP1 mediated intracellular lysosome localization and vesicle transport in BC cells. Although the mechanism of TUFT1-induced metastasis was not clear from this study, one possibility is that the mechanism is mediated by activation of the AKT/mTOR pathway. High-throughput differential gene screening showed that TUFT1 was associated with Rab5 and Rac1 [8]. Based on this, we hypothesized that TUFT1 may initiate vesicle transport by activating Rab5 and thereby affecting downstream Rac1 expression.

To test our hypothesis, we examined TUFT1, Rab5-GTP, and Rac1-GTP expression in TNBC tissue samples and analyzed the correlations with respect to TUFT1 expression. We also investigated the role of TUFT1, Rab5, and Rac1 in the metastasis and chemoresistance of TNBC cells, with the aim of evaluating TUFT1 as a prognostic and therapeutic target for TNBC.

## Methods

### Human specimens and cell lines

In our study, we recruited 80 patients with pathologically confirmed TNBC at Affiliated Hospital of Hebei University of Engineering, between January 2014 and December 2014. All patients with complete follow-up data were treated with anthracycline followed by taxanes chemotherapy after surgery. The study was conducted in accordance with the Declaration of Helsinki, and the protocol was approved by the Ethics Committee of Affiliated Hospital of Hebei University of Engineering.

The human TNBC cell lines MDA-MB-231 and HCC1937 were obtained from American Type Culture Collection (USA). Both cell lines were cultured in RPMI-1640 containing 10% fetal calf serum (FCS) and were incubated at 37 °C with 5% CO_2_.

### Immunohistochemistry analyses

Antibodies against TUFT1 (dilution 1:100, Abcam, UK), Rab5-GTP (dilution 1:100, NewEast Bioscience, USA), and Rac1-GTP (dilution 1:800, NewEast Bioscience, USA), were used for the immunohistochemical (IHC) analyses of the TNBC samples. The levels of TUFT1, Rab5-GTP, and RAC1-GTP were semi-quantitatively evaluated as described previously [7, 8]. The analysis was performed by two independent pathologists.

### RNA interference and plasmids

Recombinant lentiviruses encoding short-hairpin RNAs (shRNAs) specific for human TUFT1 or Rab5 were designed and prepared by GeneChem (Shanghai, China). The sequences of the shRNAs were as follows: TUFT1-shRNA#1: GGTGGAGTATTTACGGTAAAC, TUFT1-shRNA#2: GCAGTATTCATCCACGAATTC, Rab5-shRNA#1: GGAATCAGTGTTGTAGTAACT, Rab5-shRNA#2: GCAGTAGATTTCCAGGAAGCA, and scrambled (scr)-shRNA (negative control): 5′-TTCTCCGAACGTGTCACGTTT-3′. Cells were transfected with the lentiviruses according to relevant instructions. The efficiency of TUFT1 or Rab5 knockdown was assessed by real-time quantitative PCR and western blotting. A cell transfection efficiency > 80% was considered stable.

Recombinant retroviruses carrying the PLNCX2-vector or PLNCX2-TUFT1/p85α were synthesized according to the manufacturer’s instructions (Clontech). MDA-MB-231 or HCC1937 cells were infected with these retroviruses [with Polybrene, 8 μg/mL (Sigma-Aldrich)] and then selected with G418 [750 μg/mL (Calbiochem)].

### RNA extraction and quantitative real-time PCR

Total RNA was extracted using TRIzol reagent (Invitrogen) for reverse transcription according to manufacturer’s instructions. TUFT1 and Rab5 expression was examined by real-time PCR as described previously [7, 8].

### Co-immunoprecipitation and immunoblotting

Cells were collected and lysed with lysis buffer (20 mM Tris–HCl, pH 7.4, 150 mM NaCl, 1% Triton X-100, and protease inhibitors). The total protein concentration was determined with the BCA Protein Assay Kit (Pierce). Cell extracts containing 2–3 mg of protein were incubated with rabbit anti-RILPL2 antibodies for 2 h at 4 °C and then incubated with protein G Plus-agarose for 2 h. The resultant immunoprecipitates were washed 5 times with lysis buffer, separated by 10% SDS-PAGE, and then transferred to PVDF membranes for immunoblotting. Membranes were blocked with 5% milk in TBS-Tween for 1 h at room temperature. The membranes were then incubated with the primary antibody overnight at 4 °C and then incubation with the secondary antibody for 2 h at room temperature. Proteins were visualized by enhanced chemiluminescence (Amersham), and the results were quantified using Image-J (NIH). The antibodies used to detect TUFT1, Rab5, Rac1, p85α, p-p65, p65, cleaved caspase 3, and cleaved PARP1 were obtained from Abcam (Cambridge, UK). The antibodies used to detect XIAP and Survivin were obtained from Cell Signaling (Beverly, MA, USA).

### Transwell assay

A serum-free cell suspension was prepared, and the cells were counted and adjusted to 5 × 10^4^ cells/well in a 24-well plate. The culture medium in the upper chamber was carefully removed, and 100 μL of the cell suspension was added. Then, 600 μL of culture medium containing 30% FBS was added to the lower chamber. The Transwells were incubated at 37 °C for 24 h. Then, the chamber was fixed in 4% paraformaldehyde for 30 min, and the cells on the lower surface of the membrane were stained with 1–2 drops of staining solution for 1–3 min. Photographs were taken under a microscope.

### Rab5-GTP and Rac1-GTP pull-down assays

Cells were split in buffer containing 25 mM HEPES, 1% NP40, 10% glycerin, 5 mM MgCl_2_, 1 mM DTT, 100 mM NaCl, and protease inhibitors. The pyrolysate was incubated on ice for 5 min and then centrifuged for 1 min at 10,000×*g*. This post-nuclear supernatant was used for a pulldown analysis with 30 μg of GST-RBD pre-coated GSH beads. The beads and supernatant were incubated at 4 °C for 15 min. Then, the beads were washed with a buffer containing 0.01% NP40, boiled with SDS-PAGE buffer, and separated by SDS-PAGE. The proteins attached to the beads were analyzed by western blotting as described above. NSC23766 (Tocris Bioscience) was used to inhibit Rac1 activation.

### Tumor growth in nude mice

Four–six-week old female nude mice were obtained from Shanghai Lingchang Biological Technology, Ltd. (Shanghai, China). For the in vivo chemoresistance experiment, shTUFT1-MDA-MB-231 cells were injected into the flanks of the mice (10 mice/group). At the third week, each group was randomly divided into two subgroups, which were either untreated or treated with intraperitoneal injections of taxotere (10 mg kg^−1^) for 3 cycles, as previously described by Zhang et al. [12]. All animal handling and experimental protocols were approved by the Ethics Committee of Affiliated Hospital of Hebei University of Engineering.

### Statistical analysis

SPSS 23.0 software was used for statistical analyses. Student’s t test and Pearson’s correlation test were used to compare the variables. A *p* value less than 0.05 was considered significant.

## Results

### TUFT1 and Rab5-GTP expression are positively correlated and predict poor prognosis after chemotherapy in patients with TNBC

**Fig. 1:**
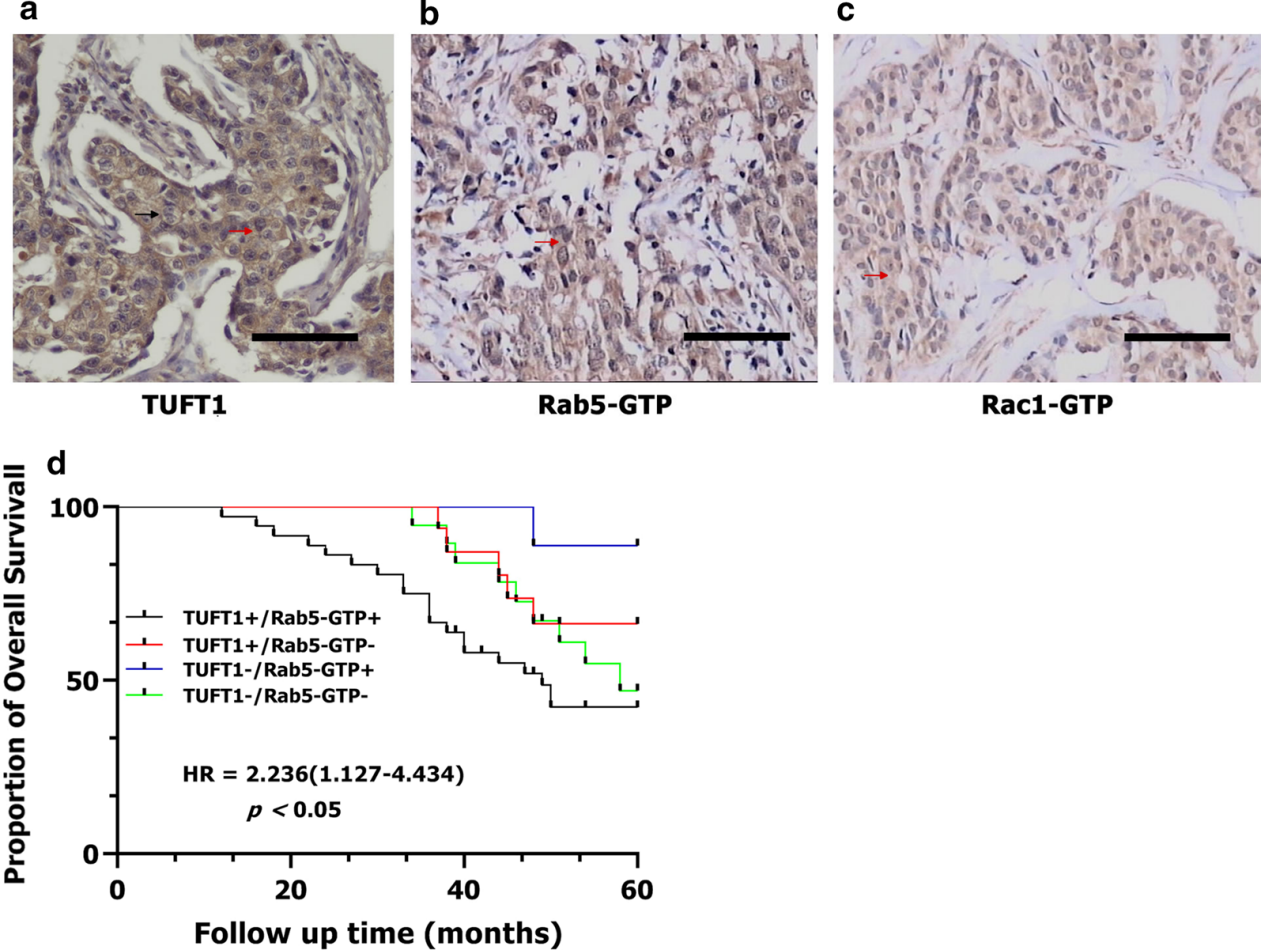
The expression of TUFT1, Rab5-GTP and Rac1-GTP in 80 TNBC patients who had received anthracycline/taxanes chemotherapy after surgery. **a**–**c** The positive expression of TUFT1, Rab5-GTP and Rac1-GTP in serial sections. Red arrows indicated cytoplasmic staining; black arrows indicated membrane staining. **d** Kaplan–Meier survival curves showing survival in 80 patients who received chemotherapy blotted in relation to TUFT1 and Rab5-GTP expression. Survival curves showing the poor overall survival in patients with tumors co-expressing TUFT1 and Rab5-GTP that received chemotherapy

In the TNBC samples, TUFT1 staining was mainly observed in the cytoplasm and cytomembrane, with the strongest staining in the cytoplasm. Rab5-GTP and Rac1-GTP staining were observed in the cytoplasm. Staining for TUFT1, Rab5-GTP, and Rac1-GTP in serial sections is presented in Fig. 1a–c. Univariate analysis showed that TUFT1 expression was positively correlated with tumor size, histological grade, axillary lymph node metastasis, and Rab5-GTP and Rac1-GTP expression (*p* = 0.024, *p* = 0.009, *p* = 0.000, *p* = 0.000, and *p* = 0.029, respectively; Table 1). We performed a logistic analysis of the above factors to exclude the effects of confounding factors. Multivariate analysis showed that Rab5-GTP and Rac1-GTP were positive associated with TUFT1 expression [Exp (B) = 6.783, *p* = 0.002 and Exp (B) = 5.522, *p* = 0.008, respectively; Table 2]. We then divided the patients into four groups according to the TUFT1 and Rab5-GTP expression levels in the TNBC samples. Follow-up analysis showed that 35 of 80 all patients died, and the 5-year overall survival rate was 56.25%. Among the patients with tumors that co-expressed TUFT1 and Rab5-GTP, 20 of 36 died, and this group displayed the lowest 5-year survival among the groups (log-rank test, *p* < 0.05, hazard ratio = 2.236 [1.127–4.434]; Fig. 1d). Therefore, TUFT1 and Rab5-GTP expression are positively correlated and predict poor prognosis in patients with TNBC after chemotherapy.

**Table 1:** The correlation between TUFT1 and clinicopathological factors in TNBC patients (n = 80)

Variable	n	TUFT1^−^	TUFT1^+^	p variable
Age				0.594
≥ 40	60	20	40	
< 40	20	8	12	
Tumor size				0.024
T1	22	12	10	
T2–3	58	16	42	
Histological grades				0.009
I, II	33	17	16	
III	47	11	36	
Lymph node metastasis				0.000
−	26	16	10	
+	54	12	42	
Rab5-GTP				0.000
−	37	21	16	
+	43	7	36	
Rac1-GTP				0.029
−	41	19	22	
+	39	9	30	

“+”, positive; “−”, negative

**Table 2:** Multivariate analysis of the factors related to TUFT1 expression

Characteristic	B	Exp (B)	95% CI for Exp (B)	p variable
Rab5-GTP	1.194	6.783	1.977–23.277	0.002
Rac1-GTP	1.709	5.522	1.567–19.454	0.008
Histological grades	0.850	2.340	0.683–8.022	0.176

*CI* confidence interval

### TUFT1 promotes metastasis of TNBC cells by up-regulating Rab5 activation

**Fig. 2:**
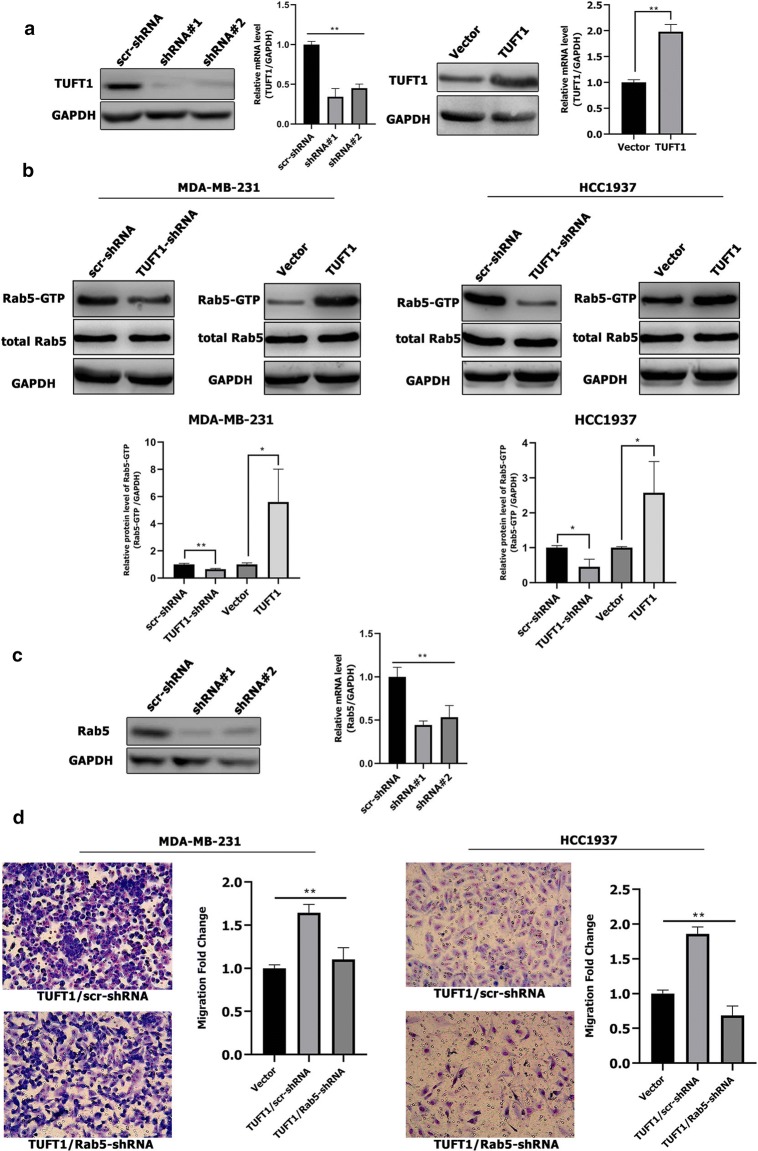
TUFT1 can regulate the Rab5 activation. **a** TUFT1 protein and mRNA expression levels were examined by western blotting and qPCR in MDA-MB-231 cells after infection with lentivirus containing TUFT1-shRNA or pLNCX2-TUFT1. **b** TUFT1 expression was downregulated by TUFT1-shRNA and upregulated by PLNCX2-TUFT1 in MDA-MB-231 and HCC1937 cells. Rab5-GTP expression was examined by western blotting (n = 3). **c** Rab5 protein and mRNA expression levels were examined by western blotting and qPCR in MDA-MB-231 cells after infection with lentivirus containing Rab5-shRNA or scr-shRNA. **d** Rab5-shRNA or scr-shRNA were transfected into TUFT1-overexpressing MDA-MB-231 and HCC1937 cells. Cell migration was monitored by transwell assay (n = 3). Results are presented as mean ± SD. The statistical significance was assessed by student’s *t* test; *p < 0.05, **p < 0.01

First, we either knocked down or overexpressed TUFT1 in MDA-MB-231 cells using shRNA. Next, we confirmed that TUFT1 protein and mRNA levels were respectively decreased and increased in TUFT1 knockdown and overexpressing cells when compared to the levels in control cells by western blotting and real-time PCR (*p* < 0.01, Fig. 2a). Then, we performed Rab5 activity assays in these cell lines, which revealed that knockdown of endogenous TUFT1 decreased Rab5-GTP levels in MDA-MB-231 and HCC1937 cells, whereas TUFT1 overexpression increased Rab5-GTP levels in both TNBC cell lines (Fig. 2b). These data indicate that TUFT1 promotes Rab5 activation in TNBC cells.

In our recently published article, we evaluated the role of TUFT1 in promoting the metastasis of TNBC cells in vivo and in vitro [13]. To investigate whether Rab5 is a primary target of TUFT1 in the regulation of TNBC metastasis, endogenous Rab5 was inhibited in TUFT1-transfected MDA-MB-231 (*p* < 0.01; Fig. 2c) and HCC1937 cells. We observed that the TUFT1-induced increase in cell migration was reversed by Rab5 knockdown in both TNBC cell lines, as assessed by transwell assays (*p* < 0.01; Fig. 2d). These results indicate that TUFT1 promotes the metastasis of TNBC cells by regulating Rab5 activation.

### Rab5 is required for TUFT1-dependent Rac1 activation in TNBC cells

**Fig. 3:**
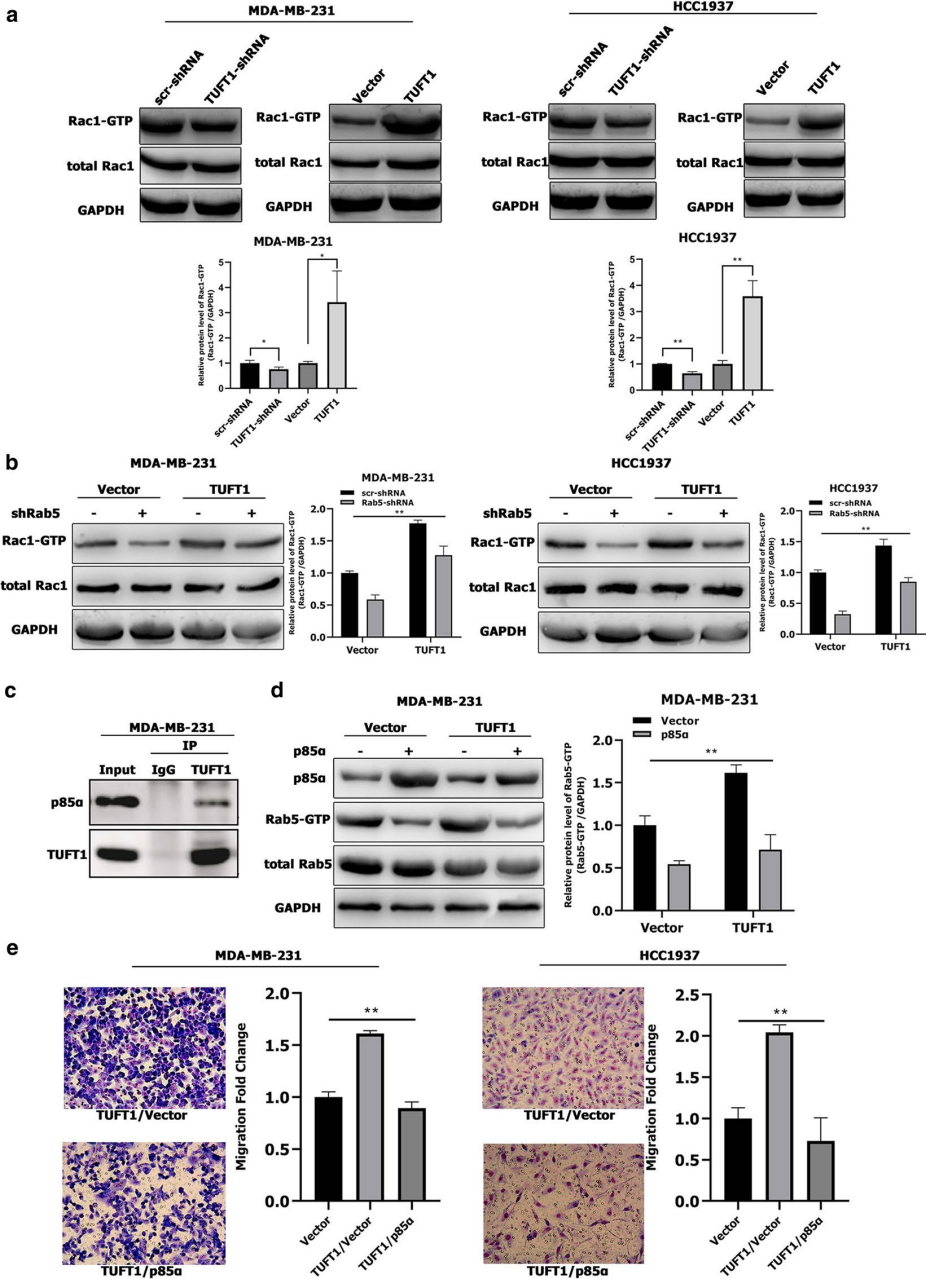
Rab5 is required for TUFT1-dependent Rac1 activation in TNBC cells. **a** TUFT1 expression was downregulated by TUFT1-shRNA and upregulated by PLNCX2-TUFT1 in MDA-MB-231 and HCC1937 cells. Rac1-GTP expression was examined by western blotting (n = 3). **b** Rab5-shRNA or scr-shRNA were transfected into TUFT1-overexpressing MDA-MB-231 and HCC1937 cells. Rac1-GTP expression was examined by western blotting (n = 3). **c** Endogenous interaction between TUFT1 and p85α in MDA-MB-231 cells by co-immunoprecipitation. **d**, **e** PLNCX2-p85α or PLNCX2-Vector were transfected into TUFT1-overexpressing MDA-MB-231 cells. **d** Rab5-GTP expression was examined by western blotting (n = 3). **e** Cell migration was monitored by transwell assay (n = 3). Results are presented as mean ± SD. The statistical significance was assessed by student’s t-test; **p < 0.01

We performed Rac1 activity assays in TUFT1-knockdown and -overexpression cells. The results of these assays revealed that knockdown of endogenous TUFT1 decreased Rac1-GTP levels in MDA-MB-231 and HCC1937 cells, whereas TUFT1 overexpression increased Rac1-GTP levels in both TNBC cell lines (Fig. 3a). These data indicate that TUFT1 promotes Rac1 activation in TNBC cells.

To investigate the potential role of Rab5 downstream of TUFT1, endogenous Rab5 was knocked down in MDA-MB-231 and HCC1937 cells transfected with either a TUFT1-expression plasmid or control vector (Fig. 3b). Rac1 activity assays revealed that Rac1-GTP levels in MDA-MB-231 and HCC1937 cells were significantly increased by TUFT1 overexpression (Fig. 3b). However, the increase in Rac1-GTP induced by TUFT1 overexpression in both TNBC cell lines was significantly decreased by Rab5 knockdown when compared to the levels in the corresponding controls (Fig. 3b). Taken together, these observations demonstrate that Rab5 is required for TUFT1-dependent Rac1 activation.

### p85α is essential for TUFT1-mediated Rab5 activation and metastasis in TNBC cells

RAB GTPase-activating protein1 (RABGAP1) can inhibit the activity of its target proteins Rab4, Rab6, Rab11, and Rab36 [14], and TUFT1 can bind to RABGAP1 to block its inhibition of Rab proteins [11]. We hypothesized that TUFT1 promotes Rab5 activation through Rab-related GAP. Therefore, we next investigated the involvement of the Rab5 GAPs, such as p85α, RabGAP5, RN-tre, Msb3, and TBC-2, in TUFT1-mediated Rab5 activation [15–19]. Strikingly, co-immunoprecipitation assays revealed that p85α was present in a complex with TUFT1 in MDA-MB-231 cells (Fig. 3c). Up-regulation of p85α substantially reduced the ability of TUFT1 to increase Rab5-GTP levels (*p* < 0.01, Fig. 3d). Accordingly, up-regulation of p85α also prevented TUFT1-dependent migration of TNBC cells (*p* < 0.01, Fig. 3e). Therefore, we concluded that the combination of TUFT1 and p85α blocked the inhibitory effect of p85α on Rab5 and enhanced TNBC cell metastasis.

### Rac1 is required for the TUFT1-mediated NF-κB pathway and activation of its downstream factors in TNBC cells

**Fig. 4:**
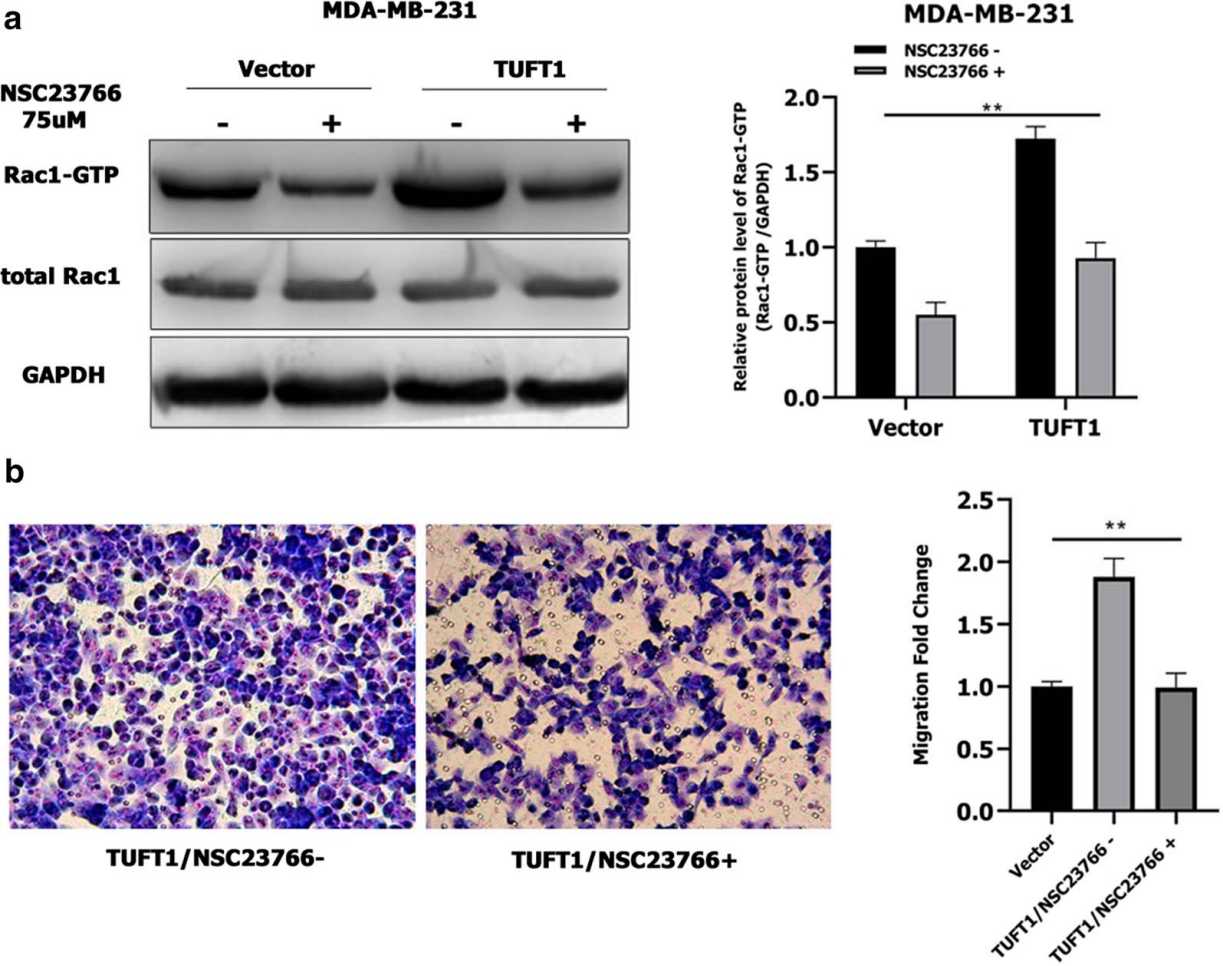
Tiam1 is involved in the activation of Rac1 by TUFT1 in TNBC cell lines. **a**, **b** TUFT1-overexpressing MDA-MB-231 cells treated with or without NSC23766. **a** Rac1-GTP expression was examined by western blotting (n = 3). **b** Cell migration was monitored by transwell assay (n = 3). Results are presented as mean ± SD. The statistical significance was assessed by student’s t-test; **p < 0.01

We further investigated the mechanism of TUFT1-induced signaling using NSC23766, an inhibitor of the Rac1 guanylate exchange factor (GEF) Tiam1 [19]. Treatment with NSC23766 decreased the Rac1-GTP levels induced by TUFT1 overexpression in MDA-MB-231 cells (*p* < 0.01, Fig. 4a) and prevented metastasis in TUFT1-transfected TNBC cells (*p* < 0.01, Fig. 4b), indicating that Tiam1 is the GEF involved in the activation of Rac1 by TUFT1 in TNBC cell lines.

**Fig. 5:**
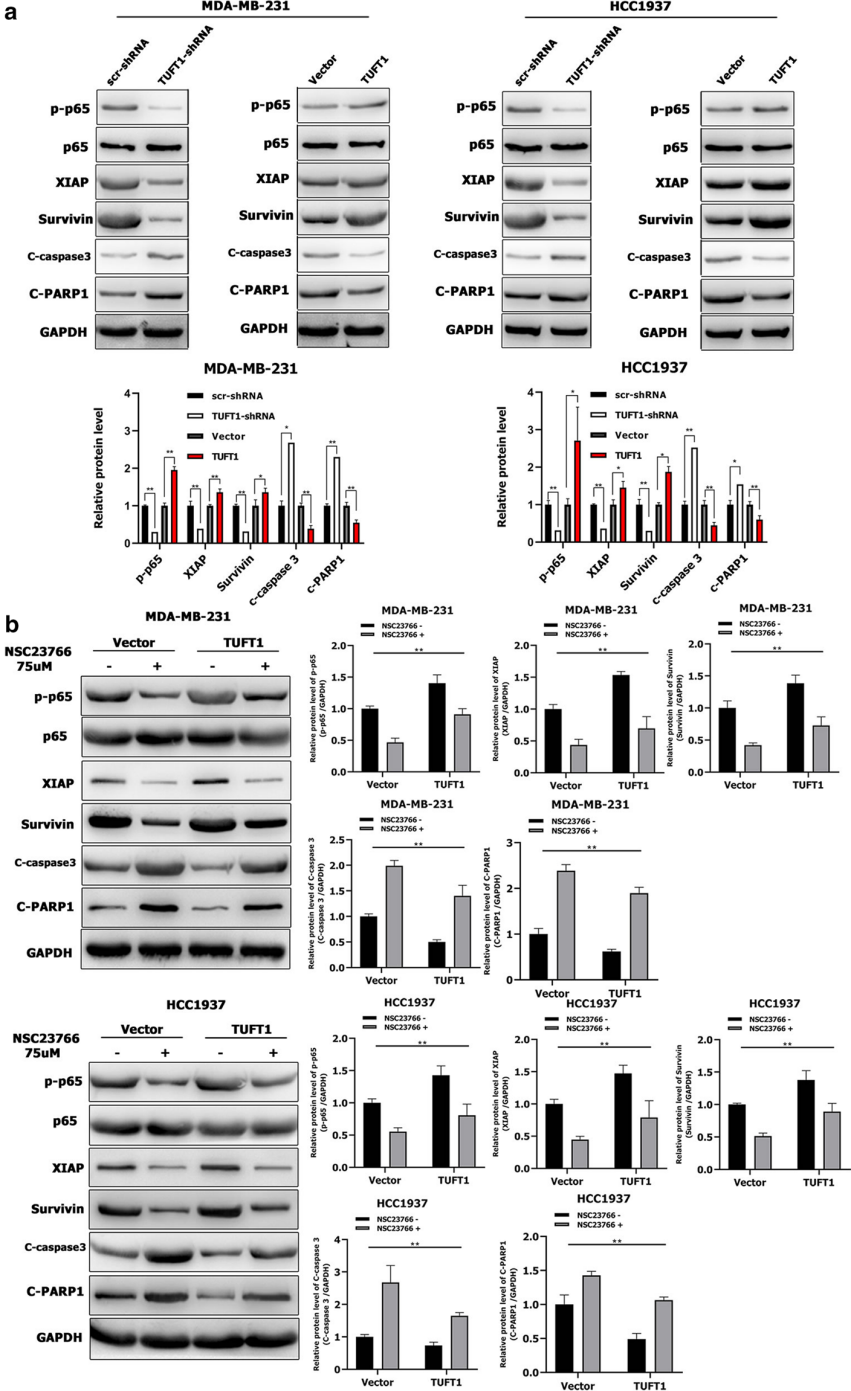
Rac1 is required for TUFT1-mediated NF-κB pathway and its downstream factors activation in TNBC cells. **a** TUFT1 expression was downregulated by TUFT1-shRNA and upregulated by PLNCX2-TUFT1 in MDA-MB-231 and HCC1937 cells. Protein levels of p-p65, p-65, XIAP, Survivin, cleaved caspase 3 and cleaved PARP1 were examined by western blotting (n = 3). **b** TUFT1-overexpressing MDA-MB-231 and HCC1937 cells treated with or without NSC23766. Protein levels of p-p65, p-65, XIAP, Survivin, cleaved caspase 3 and cleaved PARP1 were examined by western blotting (n = 3). Results are presented as mean ± SD. The statistical significance was assessed by student’s t-test; **p < 0.01

Rac1 can activate the NF-κB pathway [20], which regulates many target genes, such as XIAP and Survivin [21, 22]. XIAP and Survivin directly inhibit the activity of the apoptotic protein caspase 3, which is important for apoptosis and activation of poly (ADP ribose) polymerase (PARP) [23, 24]. In previous studies, we found that TUFT1 inhibited apoptosis and regulated the gene expression of p65 and caspase 3 in BC cells [8]. Here, we found that knockdown of TUFT1 decreased p-p65, XIAP, and Survivin levels and increased cleaved caspase 3 and cleaved PARP1 levels in MDA-MB-231 and HCC1937 cells (Fig. 5a), whereas TUFT1 overexpression increase p-p65, XIAP, and Survivin levels and decreased cleaved caspase 3 and cleaved PARP1 levels in MDA-MB-231and HCC1937 cells (Fig. 5a). These data indicate that TUFT1 promotes activation of the NF-κB pathway in TNBC cells.

Next, we inhibited Rac1 activation in TUFT1-transfected MDA-MB-231 and HCC1937 cells by treating the cells with NSC23766. Strikingly, we found that treatment with NSC23766 decreased p-p65, XIAP, and Survivin levels and increased c-caspase 3 and c-PARP1 levels in TUFT1-overexpressing TNBC cells (*p* < 0.01, respectively, Fig. 5b). Taken together, these observations demonstrate that Rac1 is required for the TUFT1-mediated NF-κB pathway and activation of proapoptotic factors.

### TUFT1 promotes chemoresistance in TNBC cells by targeting the Rab5/Rac1/NF-κB signaling pathway

**Fig. 6:**
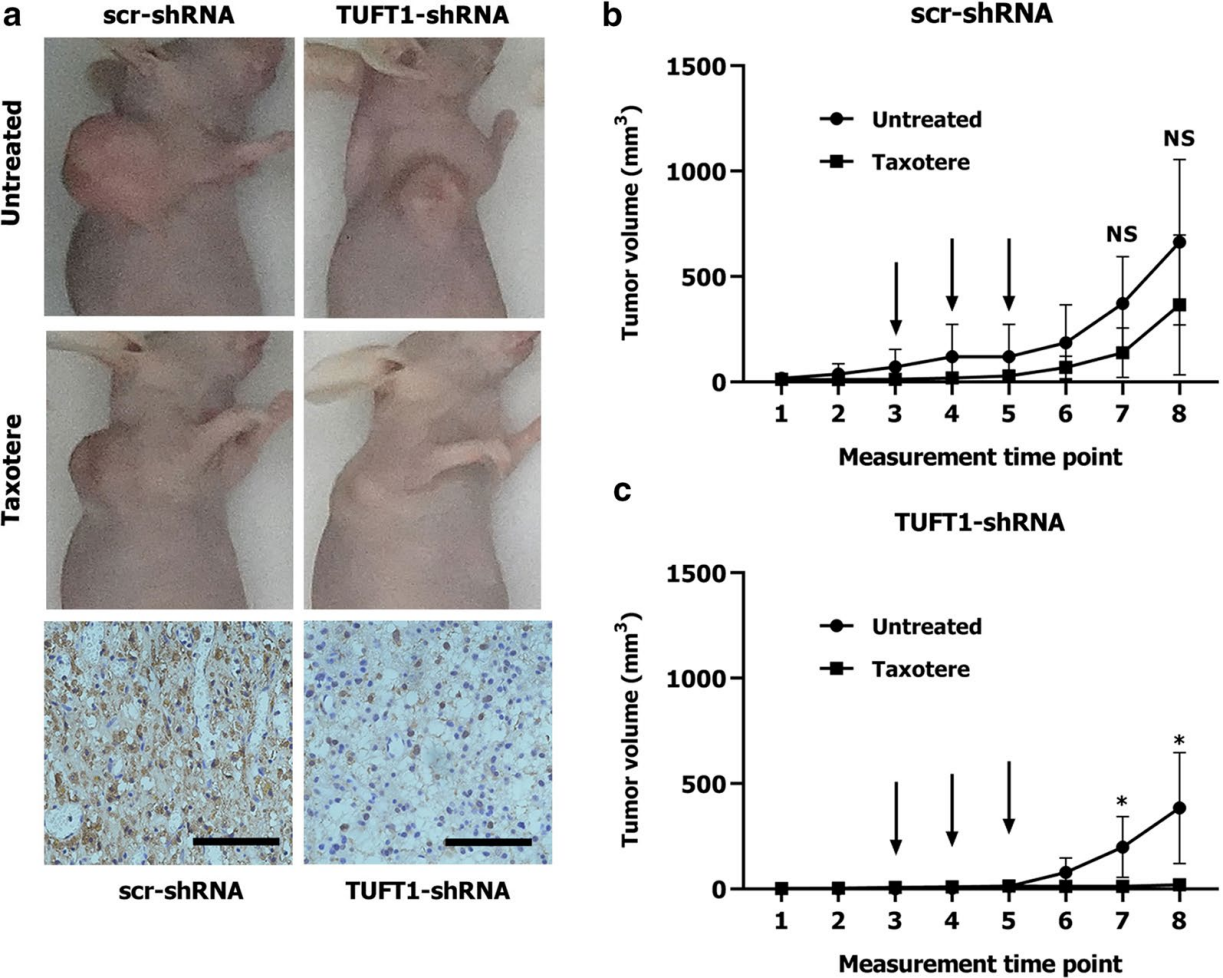
TUFT1-knockdown TNBC cells are more sensitive to taxotere in vivo. **a** scr-shRNA- and TUFT1-shRNA-MDA-MB-231 cells were injected into nude mice as described in the Materials and Methods. The tumor volumes were measured following treatment with or without Taxotere (n = 5). Representative images showing tumor formed in nude mice after injection with scr-shRNA- or TUFT1-shRNA cells and IHC staining of TUFT1 in tumor tissues (scale bars, ×400). **b**, **c** Tumour volumes in four groups. The arrows showed the time of Taxotere injection. Results are presented as mean ± SD. The statistical significance was assessed by student’s t-test; *p < 0.05

TNBC is usually highly malignant, and no effective targeted drugs are available. Therefore, chemotherapy is the main treatment for TNBC [2]. Because TUFT1 can promote proliferation and metastasis and inhibit apoptosis in MDA-MB-231 cells [8, 11], to evaluate whether TUFT1 expression directly contributes to chemotherapy resistance in TNBC, we used MDA-MB-231-shTUFT1 cells and control MDA-MB-231 cells to generate a xenograft tumor model. IHC staining revealed that the tumors formed by MDA-MB-231-TUFT1-shRNA cells had lower TUFT1 expression than the tumors formed by control cells (Fig. 6a). The size of the tumors formed by control cells was slightly reduced by taxotere treatment (*p* > 0.05, Fig. 6b), whereas the size of the tumors formed by TUFT1-shRNA cells was significantly reduced by taxotere treatment (*p* < 0.05, Fig. 6c). These results show that TUFT1 expression is directly related to the increase in chemoresistance in TNBC.

**Fig. 7:**
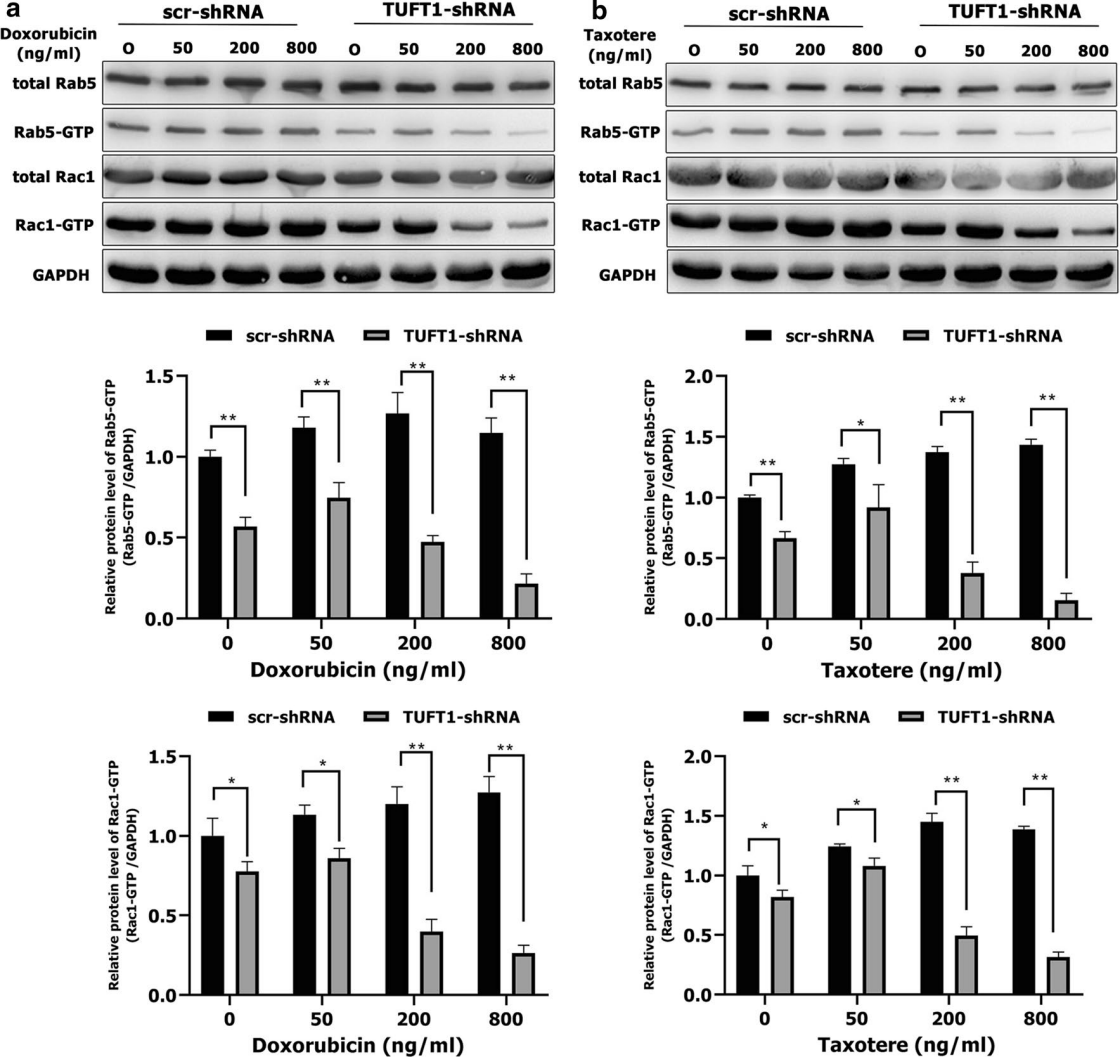
TUFT1 promotes TNBC cell resistance to chemotherapy by upregulating the Rab5/Rac1 pathway. **a**, **b** MDA-MB-231 cells were transfected with TUFT1-shRNA or scr-shRNA following treatment with various doses of doxorubicin and Taxotere for 24 h. Levels of Rab5, Rab5-GTP, Rac1 and Rac1-GTP were examined by western blotting in MDA-MB-231 cells (n = 3). Results are presented as mean ± SD. The statistical significance was assessed by Student’s t-test; **p < 0.01

**Fig. 8:**
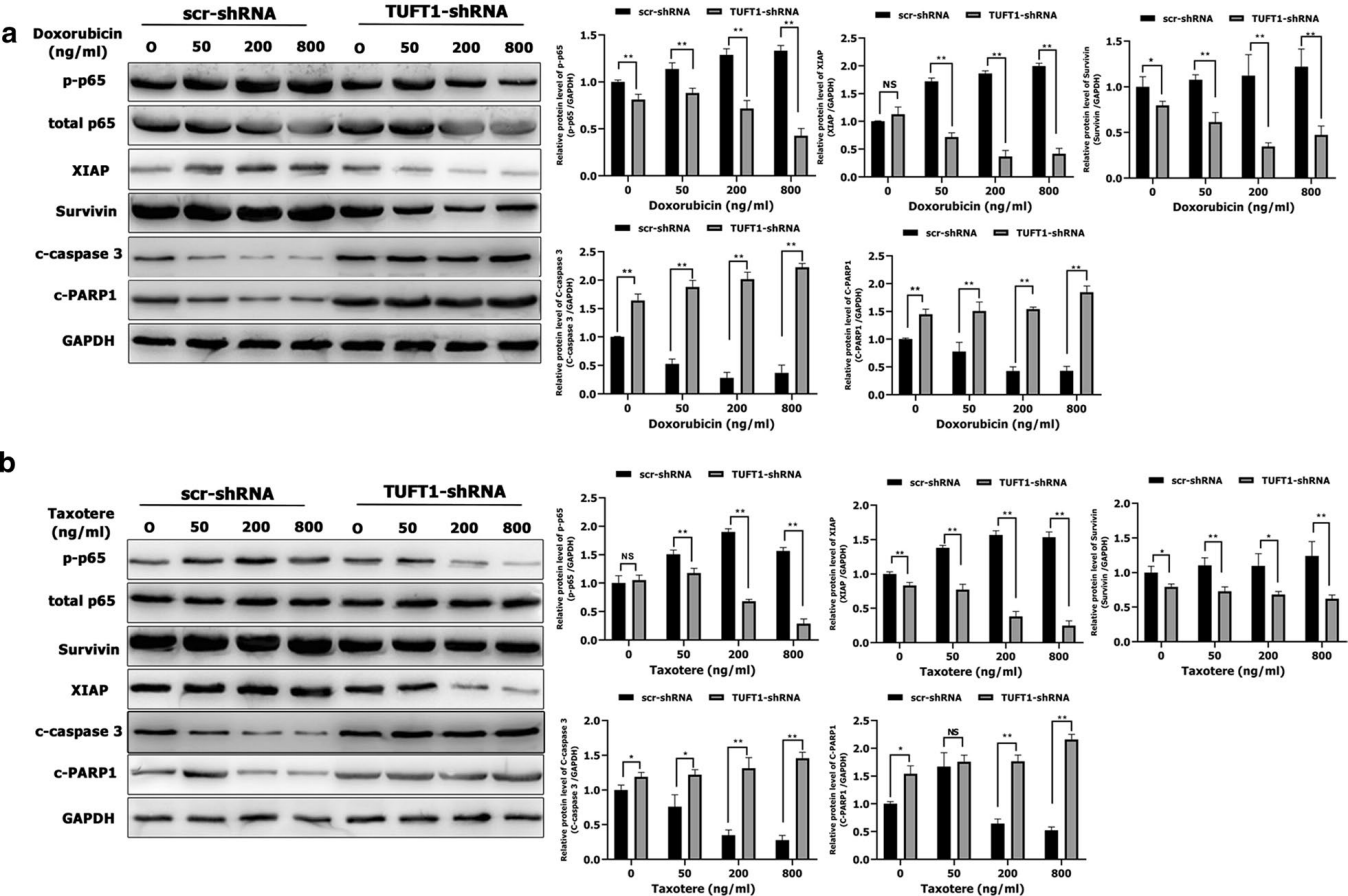
TUFT1 promotes TNBC cell resistance to chemotherapy by upregulating the NF-κB pathway. **a**, **b** MDA-MB-231 cells were transfected with TUFT1-shRNA or scr-shRNA following treatment with various doses of doxorubicin and Taxotere for 24 h. Levels of p-p65, p65, XIAP, Survivin, c-caspase 3 and c-PARP1 were examined by western blotting in MDA-MB-231 cells (n = 3). Results are presented as mean ± SD. The statistical significance was assessed by Student’s t-test; *p < 0.05, **p < 0.01

We next sought to determine whether TUFT1 confers resistance to chemotherapy in TNBC cells via the Rab5/Rac1/NF-κB signaling pathway. Treatment of TUFT1-knockdown MDA-MB-231 cells with doxorubicin and taxotere induced a decrease in both Rab5-GTP and Rac1-GTP levels in a dose-dependent manner (Fig. 7a, b). In addition, Rab5-GTP and Rac1-GTP protein levels were significantly lower in TUFT1-knockdown cells than in control cells following treatment with the corresponding doses of doxorubicin and taxotere (*p* < 0.01, Fig. 7a, b). However, the levels of total Rab5 and Rac1 protein were unchanged (Fig. 7a, b). The protein levels of p-p65, XIAP, and Survivin were significantly lower in TUFT1-knockdown cells than in control cells following treatment with corresponding doses of doxorubicin and taxotere (*p* < 0.05, Fig. 8a, b). However, the protein levels of c-caspase 3 and c-PARP1 were significantly higher in TUFT1-knockdown cells than in control cells following treatment with the corresponding doses of doxorubicin and taxotere (*p* < 0.05, Fig. 8a, b). These results indicate that TUFT1 may confer resistance to chemotherapy in BC cells by inhibiting apoptosis and proapoptotic protein activation via the Rab5/Rac1/NF-κB signaling pathway.

## Discussion

To our knowledge, this is the first systematic study on the mechanism of TUFT1-mediated metastasis and chemoresistance in TNBC. Compared to cells of other BC subtypes, basal mesenchymal-like TNBC cells display increased migration, invasion, and metastatic potential [25]. Zhou et al. [5] reported that TUFT1 overexpression promoted the metastasis of pancreatic cancer cells. They suggested that TUFT1 may affect HIF1 by influencing the expression of components of the Snail signaling pathway, which regulates epithelial-mesenchymal transition. Kawasak et al. [11] found that TUFT1 can be activated by the AKT/mTOR pathway to regulate tumor proliferation and metastasis. Our data showed that the active forms of Rab5 and Rac1 were positively associated with TUFT1 expression in TNBC samples and that co-expression of TUFT1 and Rab5-GTP predicts poor prognosis in TNBC after chemotherapy. This indicates that TUFT1, Rab5, and Rac1 may play synergistic roles in the development of TNBC.

Rab5 is also associated with the metastasis of many cancer cell lines by regulating different stages of vesicle transport [9, 26]. Rac1 plays a role in cell proliferation, apoptosis, movement, and adhesion. Rac1 also plays an important role in tumor metastasis [27]. Tiam1 is a Rac1-specific GEF that can bind to Rac1 and activate it under external stimulation, and it affects the invasion and metastasis of tumors [28]. Díaz et al. found that activated Rab5 can recruit Tiam1 to the endosome, thus promoting Rac1 activation and stimulating local actin remodeling and cell migration, leading to invasion and metastasis [19]. Our results showed that up- and down-regulation of TUFT1 have direct effects on Rab5 activation and that TUFT1-dependent cell migration is impaired in TNBC cells treated with Rab5-specific shRNA. Overexpression of TUFT1 in TNBC cells significantly up-regulated Rac1-GTP levels, and this effect was reduced by Rab5-specific shRNA. These results demonstrate that Rab5 is required for TUFT1-dependent Rac1 activation and cell metastasis. We then explored how TUFT1 regulates Rab5 activation, and we found that p85α is a GAP that can promote the hydrolysis of Rab5-GTP [19]. TUFT1 interacted with p85α to form a complex that reversed the inhibitory effect of p85α on Rab5 activation, and overexpression of p85α reduced Rab5 activation and cell metastasis induced by TUFT1. This shows that the combination of TUFT1 and p85α reverses the inhibitory effect of p85α on Rab5 and enhances TNBC cell metastasis. Furthermore, we found that TUFT1 regulated the Rac1-mediated NF-κB pathway and pro-apoptotic protein activation. However, inhibition of Rac1 activation using the Tiam1 inhibitor NSC23766 reversed this process. This is consistent with our previous studies on TUFT1 downstream-related pathway genes and our protein detection results [8]. Therefore, we believe that TUFT1 can activate the Rab5/Rac1 pathway by binding to p85α, thereby activating the downstream NF-κB pathway and ultimately promoting metastasis and inhibiting apoptosis in TNBC cells.

At present, chemotherapy is the most effective treatment for TNBC. In 2015, experts at St. Gallen agreed to recommend anthracyclines and taxanes as the main adjuvant chemotherapeutic drugs for TNBC [29]. However, more than 50% of TNBCs are resistant to adjuvant chemotherapy. Because of this chemotherapeutic resistance, relapse and metastasis are common [30]. The mechanism of Rab5- and Rac1-mediated chemoresistance has been studied in several tumors [31, 32]. Here, we demonstrated that TUFT1 knockdown can reverse taxotere resistance in a TNBC xenograft tumor model. TUFT1 knockdown conferred sensitivity to chemotherapy and increased cell apoptosis and apoptotic protein activation by downregulating the Rab5/Rac1/NF-κB signaling pathway in TNBC cells.

## Conclusion

**Fig. 9:**
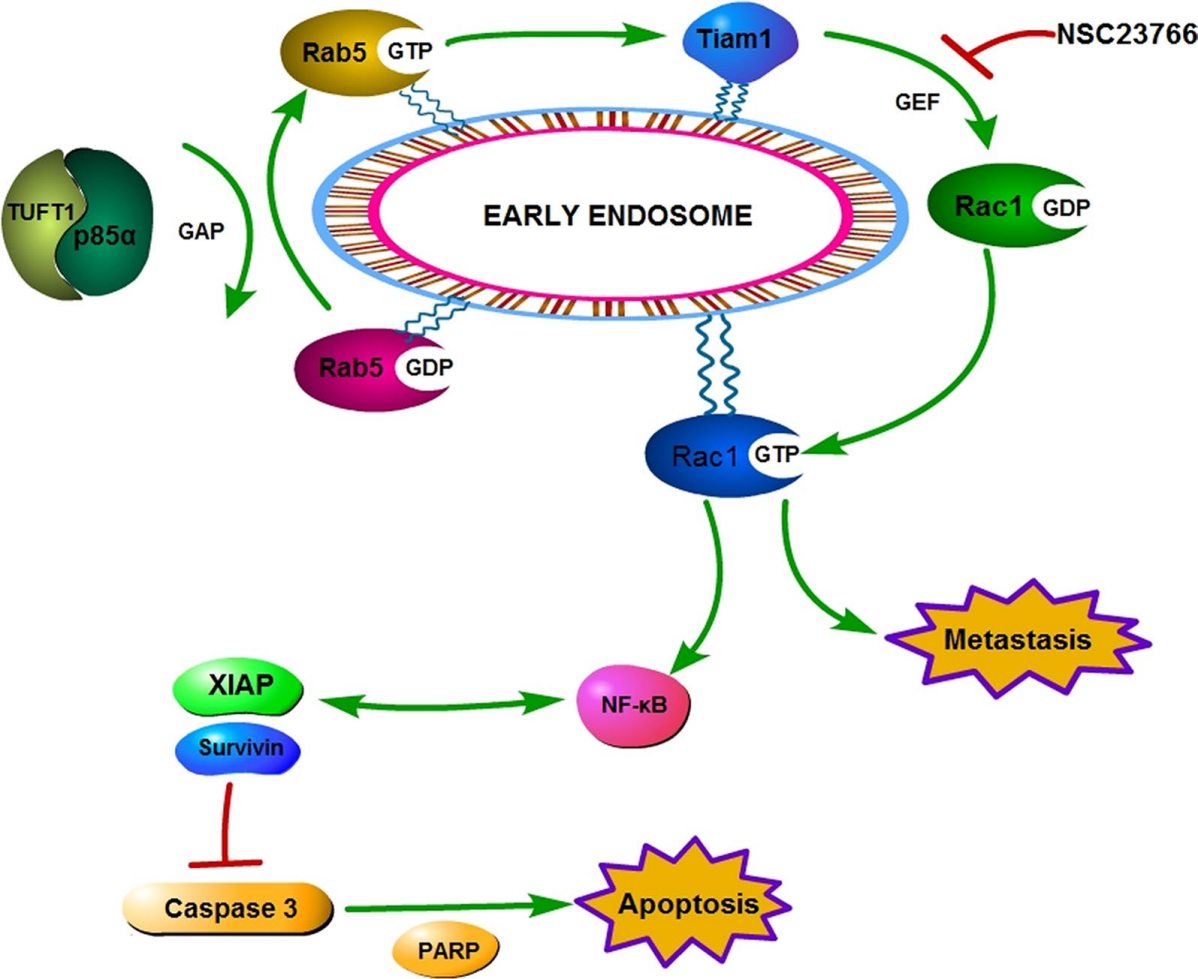
The signaling pathway proposed in this study

In summary, TUFT1 binding to p85α activates Rab5, which recruits Tiam1 to the endosomes, resulting in local activation of Rac1, activation of the downstream NF-κB pathway, and inhibition of apoptotic protein activation, ultimately promoting metastasis and chemoresistance in TNBC cells (Fig. 9). This study revealed, for the first time, the role of the TUFT1/Rab5/Rac1 signal pathway in TNBC, providing a theoretical basis for the mechanism of TNBC metastasis and chemoresistance and suggesting a new therapeutic drug target for TNBC.

## Data Availability

Datasets supporting the conclusions of this article are included within the article.
